# Predictors of the Availability of Long-Acting Injectable Antipsychotics in US Mental Health Facilities Serving Older Adults

**DOI:** 10.7759/cureus.60095

**Published:** 2024-05-11

**Authors:** Tajudeen O Basiru, Fabian Ogala, Charles Nnamchi, Oluwaseun Sonola, Sochima Egbeocha, Leroy Williams, Riveron Moises, Salisu Aikoye

**Affiliations:** 1 Behavioral Health, Community Health of South Florida, Miami, USA; 2 Behavioral Health, Houston Behavioral Healthcare Hospital, Houston, USA; 3 Crisis Intervention, Cornwall Community Hospital, Cornwall, CAN; 4 Psychiatry, American University of Integrative Sciences, Bridgetown, BRB; 5 Psychiatry, Medical University of the Americas, Charlestown, KNA; 6 Psychiatry, Charles R. Drew University of Medicine and Science, Los Angeles, USA

**Keywords:** availability, substance use facilities, mental health facilities, antipsychotics, long-acting injections

## Abstract

Introduction: Older adults are at increased risk of psychotic symptoms and even more at risk of medication nonadherence due to various factors specific to their age including memory impairment. This study aimed to examine the availability of long-acting injectable antipsychotics (LAIs) in US mental health (MH) facilities that serve older adults.

Methods: This study includes 1,216 MH facilities, using the 2022 National Substance Use and Mental Health Services Survey (N-SUMHSS) data from the Substance Abuse and Mental Health Services Administration (SAMHSA). Descriptive statistics were used to evaluate the availability of LAIs in US MHs that provide services to older adults while logistic regression was used to compare facilities that offer the services compared to those that do not.

Results: Of the total facilities included, 420 (35%) and 58 (4.8%) offered at least one LAI and all LAIs, respectively. Hospital-based facilities compared to community-based facilities, facilities that provided only MH services compared to those providing SU and MH services, facilities that offer special Alzheimer’s programs compared to those that do not, and facilities in Midwest states compared to those in East South Central, New England, and Mountain regions of the US were more likely to offer at least one LAI. Facilities that offer special services for veterans were less likely to have all LAIs examined. Only 43% of the facilities were certified by the Joint Commission.

Conclusion: Less than half of US MH facilities that serve older adults have at least one LAI service despite the usefulness of these medications in the studied population.

## Introduction

There is evidence supporting the clinical value of long-term antipsychotic use despite the unwanted effects associated with long-term use [1]. A 20-year follow-up study of over 60,000 adults with schizophrenia showed that long-term antipsychotic use was associated with significantly lower mortality compared to no antipsychotic use [2]. Both older and younger adults benefit from long-term antipsychotic use. In a study of the US prescription database, the proportion of older adults and younger adults who were prescribed antipsychotics were similar; meanwhile, approximately half of the older adults who were prescribed antipsychotics were on long-term use, indicating the utility of the medication among older adults [3]. 

Older adults have been shown to be vulnerable to a variety of medical and psychiatric conditions due to factors associated with their ages [4]. These factors, in addition to changes in cognition associated with old age, make medication adherence more difficult in this population. Nonadherence to psychotropic medications has been shown to lead to exacerbation of mental illnesses, adverse outcomes, economic burden, overutilization of healthcare resources, and less responsiveness to subsequent treatments [5,6]. Noncompliance among the elderly reportedly varies between 26% and 59% [7]. Improved availability and access to long-acting antipsychotics in US substance use and mental health facilities will likely lead to improved psychiatric outcomes among older adults. There is little research on the availability of long-acting injectable antipsychotic (LAI) antipsychotics in US mental health and substance use treatment facilities. This study therefore aimed to determine the availability and predictors of LAI antipsychotics in US facilities serving older adults.

## Materials and methods

The data for this study comes from the 2022 National Substance Use and Mental Health Services Survey (N-SUMHSS) and data is managed by the Substance Abuse and Mental Health Services Administration (SAMHSA). N-SUMHSS provides information about the number and characteristics of public and private-owned mental health and substance use facilities in the United States. Prior to 2021, facilities reported data on services via the National Survey of Substance Abuse Treatment Services (N-SSATS) and the National Mental Health Services Survey (N-MHSS). The N-SSATS and the N-MHSS were combined in 2021 and became the N-SUMHSS. In the 2022 N-SUMHSS data collection, 25,065 facilities across the United States, its territories, and the District of Columbia were surveyed which included psychiatric hospitals, general hospitals, state hospitals, community facilities, partial hospitalization/day treatment facilities, etc. Data were collected via one of the following: a web-based questionnaire, a paper questionnaire sent by mail, and a computer-assisted telephone interview (CATI). During data collection, contract personnel were available by telephone to answer any questions that the facilities may have in completing the questionnaires. Military mental health facilities, individual/small group private practices not licensed as substance use/mental health centers, and jails/prisons were excluded. Quality checks were conducted through a manual review of all completed mail questionnaires. An automated quality assurance reviews were also conducted on all survey response data after data entry.

In preparing the data for analysis, stratification was done to exclude data from facilities that do not provide services to older adults (younger than 65 years). To do this, we extracted a subset of the data using the variable that asked the question “What age groups are accepted for treatment at this facility?” To determine the facilities that offer LAI antipsychotics, we analyze the variable that asks the question “Which of the following antipsychotics are used for the treatment of serious mental illness (SMI) at this facility, at this location?” In the questionnaire, the question was accompanied by a list of all available antipsychotic medications, tabbed against their formulations (e.g., oral, LAI, etc.). A complete description of the methodologies of data collection and processing is available on the SAMHSA website [8].

Facilities that meet the criteria for this study were 1,216 substance use and mental health facilities. Descriptive statistics were used to describe facilities’ characteristics including facility types, ownership, focus (mental health vs substance use or both), government funding, availability of special programs for Alzheimer's/dementia, availability of special programs for veterans, as well as location of the facilities. Logistic regression was used to compare facilities that offer the services compared to those that do not. Outcome variables were the availability of at least one LAI service and availability of all LAI services, while predictor variables included facility characteristics described above. The most common LAI antipsychotics reported included aripiprazole, chlorpromazine, fluphenazine, paliperidone, perphenazine, olanzapine, and risperidone.

## Results

A total of 1,216 substance use and mental health facilities were included in this study, of which 420 (35%) and 58 (4.8%) offered at least one LAI and all LAIs, respectively. Most of the facilities offered both mental health and substance use treatment services (1,042 [86%]), were private nonprofit/publicly owned (965 [79%]), accepted any government funding (687 [56%]), and accepted Medicare (1,010 [83%]) and Medicaid (1,077 [89%]). Only 43% (522) of the facilities were accredited by the Joint Commission. About a third of the facilities (413 [34%]) were outpatient/partial hospitalization settings, another third (395 [32%]) were community centers, while 15% (185), 8.9% (108), and 4.7% (57) were hospital, Veteran Affairs medical centers, and residential facility settings, respectively. Close to two-thirds (757 [62%]) offered special programs for veterans, 38% (464) had supplemental employment services, 43% (524) offered housing services, and 62% (749) accepted little or no pay for services.

In the logistic regression, hospital-based facilities compared to community-based facilities (aOR [95% CI] = 2.65 [1.72, 4.08], p-value < 0.001) and facilities that offer special Alzheimer’s programs compared to those that did not (aOR [95% CI] = 1.71 [1.26, 2.33], p-value = 0.001) were more likely to offer at least one LAI. Facilities that provided both substance use and MH services compared to those providing only MH services (aOR [95% CI] = 0.60 [0.42, 0.85], p-value = 0.004) and facilities in East South Central (aOR [95% CI] = 0.49 [0.28, 0.84], p-value = 0.010), Mountain (aOR [95% CI] = 0.52 [0.30, 0.86], p-value = 0.012), and New England (aOR [95% CI] = 0.46 [0.26, 0.80], p-value = 0.007) regions of the United States compared to those in the Midwest were less likely to offer at least one LAI (Table 1). Meanwhile, facilities that offered special services for veterans were less likely to have all LAIs reported (aOR [95% CI] = 0.36 [0.18, 0.64], p-value = 0.001).

**Table 1:** Adjusted associations between facility-level characteristics and availability of LAIs. LAI: long-acting injectable; AOR: adjusted odds ratio; 95% CI: 95% confidence interval; ref: reference

Characteristics	At least one LAI					All four LAIs		
	AOR	95% CI			p-Value	AOR	95% CI	p-Value
Facility type (ref)								
Hospital-based	2.65		1.72-4.08	0.001		0.88	0.33-2.20	0.790
Multi-setting, others	0.90	0.46-1.69			0.743	1.19	0.32-3.56	0.778
Outpatient/partial hospitalization	0.84	0.60-1.19			0.337	0.51	0.22-1.10	0.093
Residential	1.41	0.71-2.74			0.316	0.93	0.13-3.86	0.928
VHA	1.30	0.72-2.35			0.377	1.87	0.48-6.48	0.343
Ownership (ref: private for profit)								
Private nonprofit/public	1.07	0.73-1.58			0.721	1.41	0.55-3.97	0.489
Focus (ref: MH services)								
Mixed MH and SU services	0.60	0.42-0.85			0.004	1.88	0.78-5.67	0.204
Accepts any government funding (ref: no)								
Yes (1)	1.27	0.91-1.78			0.154	1.26	0.62-2.69	0.531
Don't know (3)	0.55	0.33-0.89			0.016	0.46	0.10-1.52	0.250
Accepts Medicare (ref: no)								
Yes (1)	1.32	0.83-2.12			0.252	3.63	1.05-15.80	0.060
Accepts Medicaid (ref: no)								
Yes (1)	0.90	0.53-1.53			0.689	0.56	0.17-2.03	0.348
Special program for veterans (ref: no)								
Yes (1)	0.78	0.59-1.03			0.075	0.35	0.18-0.64	0.001
Offers special program for Alzheimer’s/dementia (ref: no)								
Yes (1)	1.71	1.26-2.33			0.001	1.72	0.83-3.47	0.137
US regions (ref: Midwest)								
East South Central	0.49	0.28-0.84			0.010	0.15	0.01-0.83	0.078
Mid-Atlantic	1.20	0.74-1.94			0.466	0.94	0.30-2.68	0.914
Mountain	0.52	0.30-0.86			0.012	1.59	0.62-4.01	0.325
New England	0.46	0.26-0.80			0.007	0.29	0.04-1.15	0.121
Pacific	0.87	0.52-1.43			0.583	1.04	0.33-2.98	0.943
South Atlantic	0.91	0.60-1.40			0.671	0.48	0.15-1.40	0.198
West North Central	1.88	1.06-3.35			0.031	2.42	0.87-6.56	0.083
West South Central	0.94	0.58-1.51			0.788	1.33	0.51-3.41	0.551
Emergency walk-in services (ref: no)								
Yes (1)	1.18	0.90-1.55			0.237	0.86	0.48-1.54	0.618

## Discussion

The result of this study indicates that, based on data reported to the SAMHSA, fewer than half of US substance use and mental health facilities serving older adults have at least one LAI antipsychotic. This is an important finding considering that there are many older adults who suffer from one or more schizophrenic spectrum disorders requiring long-acting antipsychotic injections. Various factors could be responsible for this finding. While studies on the availability of long-acting antipsychotic injections in US mental health facilities are lacking, there is a consensus that access to LAI antipsychotics in the United States is limited [9]. Other factors include a lack of support staff to administer antipsychotic injections in many facilities that may want to offer the service, issues with insurance coverage for LAIs, and preference by patients and their relatives for oral antipsychotics [10]. In many states, LAI antipsychotics are considered “medical” benefits which makes them more difficult to access compared to those that are considered “pharmacy” benefits which are more accessible [9]. Knowledge and willingness of providers like psychiatrists and other midlevel providers to offer patients the option of LAI may be another factor. Getzen et al. found that 55% of psychiatrists in urban centers, 25% of those in community centers, and only 10% of psychiatrists in rural centers prescribed LAI antipsychotics in their study which may suggest variations in knowledge and willingness to prescribe or availability of support service [10].

This study also found that hospital-based facilities compared to community-based ones and those that offer special Alzheimer’s disease programs compared to those that do not were more likely to offer at least one LAI antipsychotic. The former finding may be related to the fact that hospital-based facilities are more likely to have nursing services and other support staff available, making prescription and administration of LAI feasible compared to community facilities where there are possible personnel shortages [11]. Facilities that offer special programs for Alzheimer’s disease are likely to be well-funded and well-staffed and are therefore less likely to be subjected to personnel challenges [12]. For instance, Maas et al. reported an increase in the number of nursing homes with special care units for people with Alzheimer’s disease and the associated higher cost compared to traditional nursing homes [12]. They are also likely to be in urban settings where psychiatrists are more likely to prescribe LAI antipsychotics compared to rural facilities [10].

Location-wise, facilities in the mountain region of the US (comprising states of Arizona, Colorado, Idaho, Montana, New Mexico, Utah, Nevada, and Wyoming) were found to be more likely to have at least one LAI antipsychotic compared to other states [13]. However, analysis of SAMHSA data by the American Addiction Centers indicates that these states in the mountain region of the United States are not the states with the highest per capita mental health spending [14]. Therefore, factors other than funding, such as those explained above, may explain this finding. A systematic review of studies on barriers to LAI antipsychotics among schizophrenic patients identified various clinicians, patients, and system-related factors [15]. The system factors identified include lack of insurance coverage, cost associated with storage of LAIs, requirement for staff training on LAI administration, and the difficulty associated with transferring LAI administration from a psychiatrist to a primary care physician. Most of these factors tend to revolve around funding. Meanwhile, studies have shown that nonadherence to antipsychotic medications is expensive in the long term and associated with excessive utilization of healthcare resources with profound implications for human resources and burnout [16].

Limitations

The strength of this study lies in its uniqueness in that it studied the availability of long-acting antipsychotic medications in facilities that provide services to older adults in the United States given that there are very few studies that have examined this subject. Also, the study used recent data (2022) that was collected nationally using a standardized methodology. However, there are several limitations to this study including the fact that data are reported by states and variations in data collection in the different states may affect the quality of data reported to SAMHSA. Many state-specific factors, such as availability of funding, regulatory restrictions, and variation in data collection and reporting policies, may affect the quality of data collected. Another limitation is that only 88% of eligible facilities responded to the surveys. Because participation is voluntary, it is impossible to get data from every eligible facility. There were no adjustments for nonresponse facilities. This may limit the inference that could be made from the findings of this study.

## Conclusions

Our study is unique given that there are few studies that have examined the availability of LAI antipsychotics in US substance use and mental health facilities in the general population or in the geriatric population. The findings are also important given that the population of older adults in the United States is expected to grow in the coming years because of improvements in healthcare and life expectancy. Therefore, the proportion of people living with schizophrenia spectrum disorders, and so requiring lifelong antipsychotics can be expected to grow. Because studies have shown that adherence to antipsychotic medications significantly reduces the economic burden associated with schizophrenia spectrum disorders, it is paramount to improve access to LAI antipsychotics among the older adult population. This improvement is likely to enhance adherence to the medications.
